# *Lactobacillus acidophilus* Suppresses Colitis-Associated Activation of the IL-23/Th17 Axis

**DOI:** 10.1155/2015/909514

**Published:** 2015-04-20

**Authors:** Linlin Chen, Yiyou Zou, Jie Peng, Fanggen Lu, Yani Yin, Fujun Li, Junwen Yang

**Affiliations:** ^1^Department of Gastroenterology, Xiangya Hospital of Central South University, Changsha 410011, China; ^2^Department of Gastroenterology, Second Xiangya Hospital of Central South University, Changsha 410008, China

## Abstract

The aim of this paper is to determine the modulatory effects of* Lactobacillus acidophilus* on the IL-23/Th17 immune axis in experimental colitis. DSS-induced mouse models of UC were to be saline, hormones, and different concentrations of* Lactobacillus acidophilus* intervention. The expression of interleukin- (IL-) 17, tumor necrosis factor *α* (TNF*α*), IL-23, transforming growth factor *β*1 (TGF*β*1), signal transducer and activator of transcription 3 (STAT3), and phosphorylated (p)-STAT3 was examined by RT-PCR, Western blotting, and immunohistochemical analysis. And the results showed that administration of* L. acidophilus* suppressed Th17 cell-mediated secretion of proinflammatory cytokine IL-17 through downregulation of IL-23 and TGF*β*1 expression and downstream phosphorylation of p-STAT3.

## 1. Introduction

The most common inflammatory bowel diseases (IBDs) are Crohn's diseases (CD) and ulcerative colitis (UC). It is recognized that aberrant immune response in the mucosa of the gastrointestinal tract plays an important role in pathogenesis of IBDs [1, 2]. A large body of evidence from experimental models of IBD has shown that CD4+ T cells (T helper cells Th1 and Th2) play a major role in initiating and regulating the immunopathologic process, largely based on the production of proinflammatory cytokines, such as tumor necrosis factor*α* (TNF*α*) [3, 4]. More recently, another subset of T helper cells that produces the cytokine interleukin (IL-17, also called IL-17A) was identified as Th17 cells and has gained particular attention due to its proinflammatory role in the mucosal immune response [5, 6]. The number of Th17 cells and IL-17 expression were found to be significantly enhanced in the inflamed gut of CD and UC patients [7, 8]. In addition to the IL-17 isoforms (IL-17A and IL-17F), Th17 cells also secrete IL-6, IL-21, IL-22, and IL-26 [9]. IL-17 drives microbial defense [10], contributes to neutrophil migration and function [11], and promotes T-cell priming and cellular production of inflammatory mediators, such as IL-1, IL-6, TNF*α*, granulocyte macrophage colony-stimulating factor (GM-CSF), nitric oxide synthase (NOS)-2, prostaglandin E2, and metalloproteases [12]. Therefore, control of the differentiation and function of Th17 cells is potentially significant for IBD treatment [13].

It has been recently demonstrated that differentiation of Th17 cells is initiated by transforming growth factor *β*1 (TGF*β*1) and IL-6 [9, 14]. Both TGF*β*1 and IL-6 regulate Th17 differentiation by activating the transcription factor signal transducer and activator of transcription 3 (STAT3) [15, 16]. A line of evidence has also indicated a significant role of IL-23 in Th17 differentiation. IL-23 was initially thought to be critical for Th17 differentiation; however, it was later found to be rather important for Th17 cell expansion, stabilization, and/or conditioning for a fully inflammatory cell phenotype [17, 18]. The IL-23/Th17 immune axis has thus been shown to play a crucial role in a number of chronic inflammatory diseases including IBD [19].

Commensal* Lactobacillus* species are common inhabitants of the natural microbiota in the human gut. The* Lactobacillus* species can rebalance homeostasis in gastrointestinal inflammatory diseases and thus have a protective role against IBD. We have previously shown that* Lactobacillus (L.) acidophilus* treatment can efficiently ameliorate dextran sodium sulfate- (DSS-) induced experimental colitis in mice [20]. In the present study, we examined whether the therapeutic effect of* L. acidophilus* is achieved through suppression of the IL-23/Th17 pathway, including Th17 cell differentiation and the expansion factors IL-23, TGF*β*1, and STAT3, as well as Th17 cell-secreted cytokines IL-17 and TNF*α*. We found that oral administration of* L. acidophilus* at all doses induced a significant downregulation of colitis-enhanced expression of all the examined factors.

## 2. Materials and Methods

### 2.1. Bacterial Strain

*L. acidophilus* was isolated from a normal human intestinal tract SMC-S095 sample and sequence-verified by our laboratory [20]. After culturing under anaerobic conditions with De Man, Rogosa, Sharpe (MRS) medium for 24 h, the bacteria were collected, quantified by a spectrophotometer, and diluted with normal saline to 10^10^ CFU/mL.

### 2.2. Study Design

Seventy-two female BALB/c mice (6–8 weeks old, 20.0 ± 2.0 g mean body weight) were purchased from Hunan Agricultural University and housed under standard conditions (50% ± 10% humidity, 12 h light/dark cycle, and* ad libitum* access to standard mouse chow). Colitis was established in 64 mice by adding 5% DSS (MW 50000; Sigma Chemical Co., St. Louis, MO) to the drinking water and allowing* ad libitum* access for 7 days. The mice were randomly divided into the following control and experimental model groups (*n* = 8 each; day 0): the nontreated model group; the vehicle treated model group (oral gavage of 1 mL/10 g normal saline); the* L. acidophilus*-treated groups C4–C8 (oral gavage of 10^4^, 10^5^, 10^6^, 10^7^, or 10^8^ CFU/10 g body weight, resp.); the prednisone acetate (an anti-inflammatory agent) treated positive control (administered at 45 *μ*g/10 g body weight). Eight mice that were given regular water and received no subsequent treatments served as the normal control group.* L. acidophilus* was administered on the same day when DSS feeding was started. Different doses were applied in order to identify an appropriate condition that can efficiently modulate the expression of factors related to Th17 cell function. On posttreatment day 7, all mice were sacrificed with ether and the colon was collected. A 0.5 cm segment of the distal colon that is 1 cm proximal to the anus was excised and fixed with 10% formalin for later paraffin embedding and sectioning. The rest of the distal colon was stored in liquid nitrogen for RNA and protein extractions.

### 2.3. Reverse Transcription PCR (RT-PCR)

The colonic tissue was homogenized, and RNA was extracted by using TRIzol (Invitrogen, Grand Island, NY) according to the manufacturer's instructions. First-strand cDNA was synthesized using a Reverse Transcription Kit (MBI, Ottawa, CA). Primers for PCR analysis were obtained from Invitrogen (Shanghai, China). The primer sequences were as follows: *β*-actin forward primer 5′-TGGAATCCTGTGGCATCCATGAAAC-3′ and reverse primer: 5′-TAAAACGCAGCTCAGTAACAGTCCG-3′ (product length of 349 bp, Tm 60°C, 30 cycles), IL-17 forward primer 5′-TCAGACTACCTCAACCGTTCC-3′ and reverse primer 5′-CAGTTTCCCTCCGCATT-3′ (product length of 129 bp, Tm 54.5°C, 30 cycles), IL-23 forward primer 5′-AATAATGTGCCCCGTATCCA-3′ and reverse primer 5′-AGGCTCCCCTTTGAAGATGT-3′ (product length of 144 bp, Tm 58°C, 28 cycles), TNF*α* forward primer 5′-GGTTGTCTTTGAGATCCATGC-3′ and reverse primer 5′-ACGTGGAACTGGCAGAAGAG-3′ (product length of 411 bp, Tm 54.5°C, 30 cycles), TGF*β* forward primer 5′-GAAGTGGATCCACGAGCCCAAG-3′ and reverse primer 5′-GCTGCACTTGCAGGAGCGCAC-3′ (product length of 247 bp, Tm 60°C, 30 cycles), and STAT3 forward primer:5′-ACCCAACAGCCGCCGTAG-3′ and reverse primer 5′-CAGACTGGTTGTTTCCATTCAGAT-3′ (product length of 192 bp, Tm 62°C, 30 cycles). Multiplex reactions were run in duplicate, and PCR products were run on agarose gels. The intensity of DNA bands was measured with Quantity One software (Bio-rad, Hercules, CA), and the results were normalized to the internal control *β*-actin.

### 2.4. Western Blotting

Colon tissues were homogenized on ice in protein lysis buffer. The total homogenate was then centrifuged at 12,000 rpm for 30 minutes at 4°C. The supernatant was collected, and protein concentration was measured using the DC protein assay method (Bio-rad, Hercules, CA). Twenty *μ*g proteins were separated on SDS-PAGE gels and transferred to PVDF membranes. The membrane was then blotted with primary antibodies for 1 h at room temperature. Antibodies to IL-17 (H-132), TNF*α* (N-19), TGF*β*1 (V), STAT3 (H-190), and *β*-actin were purchased from Santa Cruz Biotechnology (Santa Cruz, CA), and anti-IL-23 (P19) and anti-p-STAT3 (Y705) antibodies were obtained from Abcam (Cambridge, UK). Antibody dilution ratios: (1) IL-17 and IL-23, 1 : 400; (2) STAT3, p-STAT3 and TGF*β*1, 1 : 500; (3) TNF*α*, 1 : 2000; and (4) *β*-actin, 1 : 5000. After three 10 min washes, membranes were incubated with anti-mouse, -rabbit, or -goat horseradish peroxidase (HRP) conjugated secondary antibodies (ZSGB-Bio, Beijing, China) and washed, and detection was achieved using a DAB kit (ZSGB-Bio). Densitometric analysis was performed using Quantity One software, and the results are expressed as the relative intensity (OD) of target protein to that of *β*-actin control.

### 2.5. Immunohistochemistry

Mouse colonic tissues embedded in paraffin blocks were cut into 4 *μ*m sections. Sections were deparaffinized, and antigen retrieval was performed by microwave treatment. Nonspecific blocking was obtained by incubation in phosphate-buffered saline (PBS) containing 5% normal goat serum for 30 minutes at room temperature. Tissue sections were then incubated with anti-p-STAT3 (1 : 200) antibody for 1 h at room temperature. Sections were washed with PBS, incubated in the ABC reagent for 1 hour at room temperature, washed again, and incubated in a peroxidase solution (ZSGB-Bio). Specimens were counterstained with hematoxylin, dehydrated, and coverslipped. The signal of immunohistochemical staining was analyzed with Image-Pro Plus 6.0 software (Media Cybernetics, Maryland, USA).

### 2.6. Statistical Analysis

All statistical analyses were carried out using the SPSS statistical software suite (version 16.0; SPSS Inc., Chicago, IL). Results are expressed as mean ± standard deviation (SD). Single-factor analysis of variance (one-way ANOVA) was used to analyze the differences between groups, with *P* < 0.05 considered as statistical significance.

## 3. Results

### 3.1. *L. acidophilus* Administration Suppressed the Expression of IL-17 and TNF*α* in Colitis

**Figure 1:**
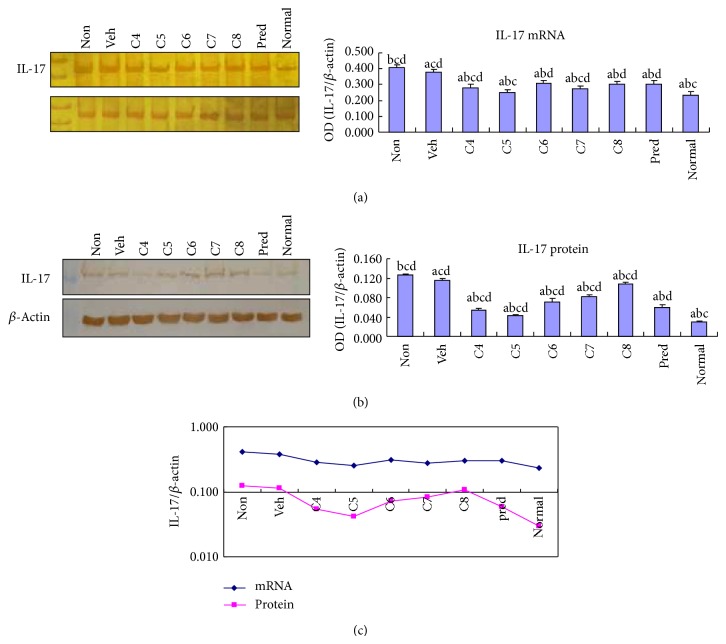
Oral administration of* Lactobacillus acidophilus* inhibited IL-17 expression in DSS-induced colitis. The mRNA (a) and protein (b) levels of IL-17 expression were examined by RT-PCR and Western blotting, respectively, in the colonic tissues of normal control mice group (normal) and DSS-induced mice groups, including* L. acidophilus*-treated groups C4–C8 (oral gavage of 10^4^, 10^5^, 10^6^, 10^7^, or 10^8^ CFU/10 g body weight, resp.), prednisone acetate treated positive control (Pred), and nontreated (Non) and vehicle (Veh) treated control groups. Representative electrophoresis images are shown on the left, and bar graphs presenting mean ± SD (*n* = 8) values are shown on the right. A plotted trendline chart shows IL-17 expression in each group at both mRNA and protein levels (c). ^a^ *P* < 0.05 versus Non, ^b^ *P* < 0.05 versus Veh, ^c^ *P* < 0.05 versus Pred, and ^d^ *P* < 0.05 versus normal.

We have recently shown that oral administration of* L. acidophilus* ameliorates the pathogenesis of colitis in mice [20]. Given the critical role of Th17 cells in autoimmune diseases including IBD, we hypothesized that* L. acidophilus* might improve colitis at least partially through suppression of Th17 cell function. We thus initially sought to examine whether colonic expression of IL-17, the hallmark cytokine of Th17 cells, is modulated by* L. acidophilus* treatment. The expression level of IL-17 was examined in all nine groups at mRNA and protein levels. We found that both mRNA and protein expression of IL-17 were significantly (*P* < 0.05) enhanced in DSS-induced colitis (Figures 1(a) and 1(b)), which is consistent with the results of a previous report [21].* L. acidophilus* application at different doses in the C4–C8 groups all significantly (*P* < 0.05) attenuated colitis-increased IL-17 expression (Figures 1(a) and 1(b)) compared to levels in the nontreated or vehicle treated groups. Of all the* L. acidophilus-*treated groups, C5 showed the greatest inhibition of IL-17 expression at both the mRNA and protein levels, and this inhibition was even superior to the therapeutic effect of prednisone treatment (Figure 1(c)). The electrophoresis images showing the mRNA and protein expression of internal control *β*-actin are presented for IL-17 expression in Figures 1(a) and 1(b) and are not repeatedly shown for the other examined molecules.

**Figure 2:**
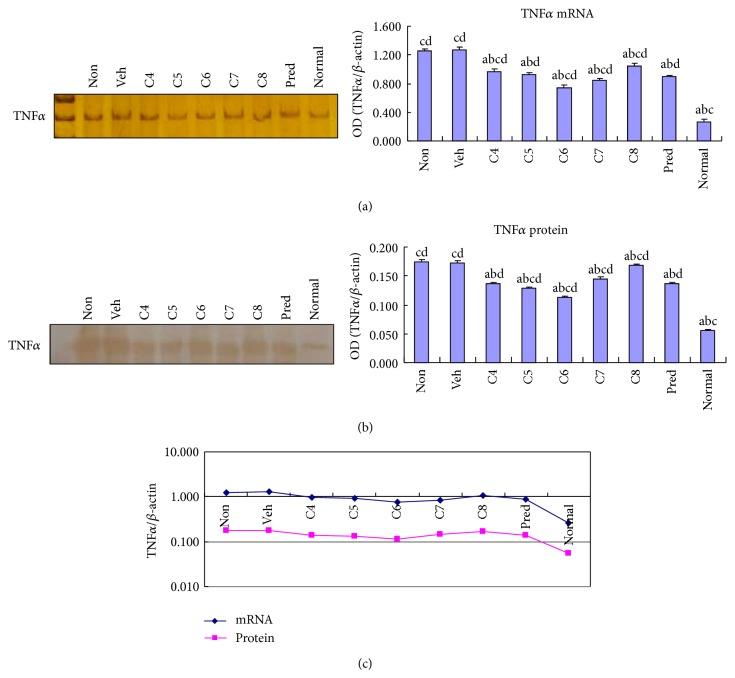
*Lactobacillus acidophilus* treatment suppressed TNF*α* expression in colitis. The mRNA (a) and protein (b) levels of TNF*α* expression were examined by RT-PCR and Western blotting, respectively, in the colonic tissues of normal control mice group (normal) and DSS-induced mice groups, including* L. acidophilus*-treated C4–C8 groups, prednisone acetate treated positive control (Pred), and nontreated (Non) and vehicle (Veh) treated control groups. Representative electrophoresis images are shown on the left, and bar graphs presenting mean ± SD (*n* = 8) values are shown on the right. A plotted trendline chart shows TNF*α* expression in each group at both mRNA and protein levels (c). ^a^ *P* < 0.05 versus Non, ^b^ *P* < 0.05 versus Veh, ^c^ *P* < 0.05 versus Pred, and ^d^ *P* < 0.05 versus normal.

Because TNF*α* is another important proinflammatory cytokine secreted by Th17 cells, we also examined the effect of* L. acidophilus* treatment on TNF*α* expression. Similarly to IL-17, TNF*α* expression at both the mRNA and protein levels was significantly (*P* < 0.05) decreased by* L. acidophilus* treatment in groups C4–C8 compared to the vehicle treated control group (Figures 2(a) and 2(b)). However, unlike IL-17, TNF*α* expression was lowest in Group C6 among all the DSS-treated groups (Figure 2(c)).

### 3.2. *L. acidophilus* Downregulated IL-23 and TGF*β*1 Expression in Colitis

**Figure 3:**
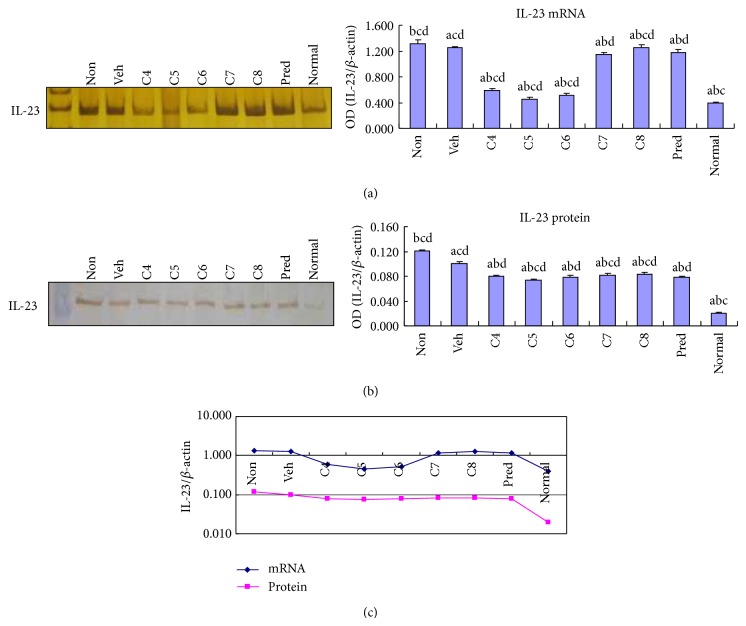
*Lactobacillus acidophilus* administration decreased IL-23 expression in colitis. The mRNA (a) and protein (b) levels of IL-23 expression were examined by RT-PCR and Western blotting, respectively, in the colonic tissues of normal control mice group (normal) and DSS-induced mice groups, including* L. acidophilus*-treated C4–C8 groups, prednisone acetate treated positive control (Pred), and nontreated (Non) and vehicle (Veh) treated control groups. Representative electrophoresis images are shown on the left, and bar graphs presenting mean ± SD (*n* = 8) values are shown on the right. A plotted trendline chart shows IL-23 expression in each group at both mRNA and protein levels (c). ^a^ *P* < 0.05 versus Non, ^b^ *P* < 0.05 versus Veh, ^c^ *P* < 0.05 versus Pred, and ^d^ *P* < 0.05 versus normal.

**Figure 4:**
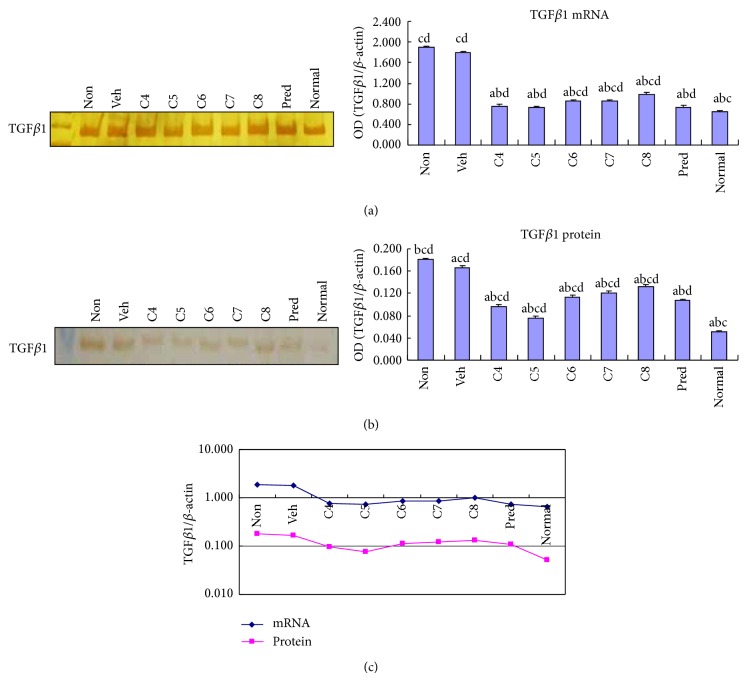
Treatment with* Lactobacillus acidophilus* reduced TGF*β*1 expression in colitis. The mRNA (a) and protein (b) levels of TGF*β*1 expression were examined by RT-PCR and Western blotting, respectively, in the colonic tissues of normal control mice group (normal) and DSS-induced mice groups, including* L. acidophilus-*treated C4–C8 groups, prednisone acetate treated positive control (Pred), and nontreated (Non) and vehicle (Veh) treated control groups. Representative electrophoresis images are shown on the left, and bar graphs presenting mean ± SD (*n* = 8) values are shown on the right. A plotted trendline chart shows TGF*β*1 expression in each group at both mRNA and protein levels (c). ^a^ *P* < 0.05 versus Non, ^b^ *P* < 0.05 versus Veh, ^c^ *P* < 0.05 versus Pred, and ^d^ *P* < 0.05 versus normal.

IL-23 and TGF*β*1 have been shown to play essential roles in promoting Th17 cell differentiation, expansion, and function [9, 14, 17, 18]. We tested whether the inhibitory effect of* L. acidophilus* on Th17-associated cytokine expression is achieved through downregulation of IL-23 and/or TGF*β*1 expression, thereby suppressing Th17 cell differentiation and stabilization. Interestingly, treatment of colitis with a lower dose of* L. acidophilus* (C4–C6 groups) caused a dramatic decrease in IL-23 mRNA expression, which reached a level comparable to that of the normal control group (Figure 3(a)). Moreover, all* L. acidophilus*-treated groups showed a significant (*P* < 0.05) decrease in IL-23 protein expression (Figure 3(b)). Notably, both the mRNA and protein levels of IL-23 expression were lowest in group C5 among all the colitis groups (Figure 3(c)), at a level similar to that observed for IL-17 expression. Likewise,* L. acidophilus* administration at all doses induced marked inhibition of TGF*β*1 mRNA expression (Figure 4(a)). A dramatic attenuation of TGF*β*1 protein expression was also observed in the low dose groups, C4 and C5 (Figure 4(b)). Again, mice treated with 10^5^ CFU/10 g* L. acidophilus*, group C5, showed the lowest expression of TGF*β*1 (Figure 4(c)).

### 3.3. *L. acidophilus* Inhibited STAT3 Expression and Phosphorylation

**Figure 5:**
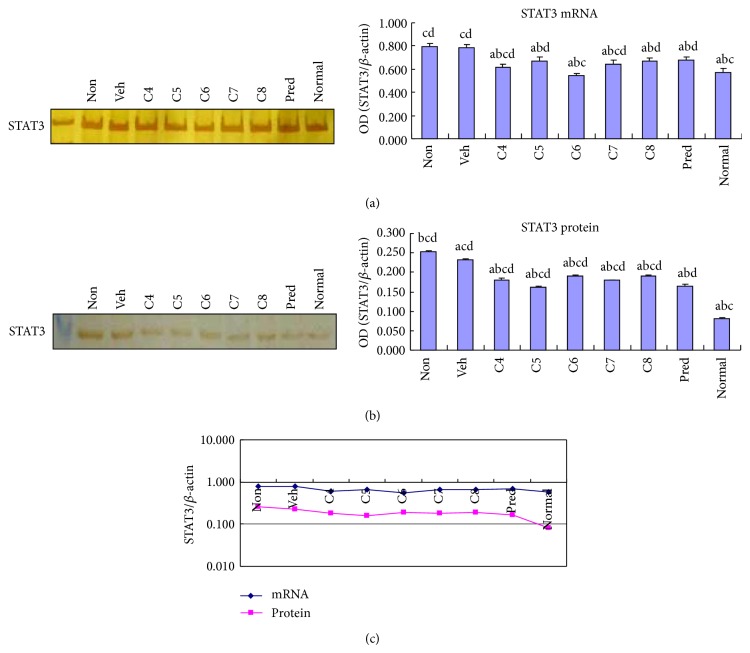
*Lactobacillus acidophilus* administration decreased STAT3 expression in colitis. The mRNA (a) and protein (b) levels of STAT3 expression were examined by RT-PCR and Western blotting, respectively, in the colonic tissues of normal control mice group (normal) and DSS-induced mice groups, including* L. acidophilus*-treated C4–C8 groups, prednisone acetate treated positive control (Pred), and nontreated (Non) and vehicle (Veh) treated control groups. Representative electrophoresis images are shown on the left, and bar graphs presenting mean ± SD (*n* = 8) values are shown on the right. A plotted trendline chart shows STAT3 expression in each group at both mRNA and protein levels (c). ^a^ *P* < 0.05 versus Non, ^b^ *P* < 0.05 versus Veh, ^c^ *P* < 0.05 versus Pred, and ^d^ *P* < 0.05 versus normal.

**Figure 6:**
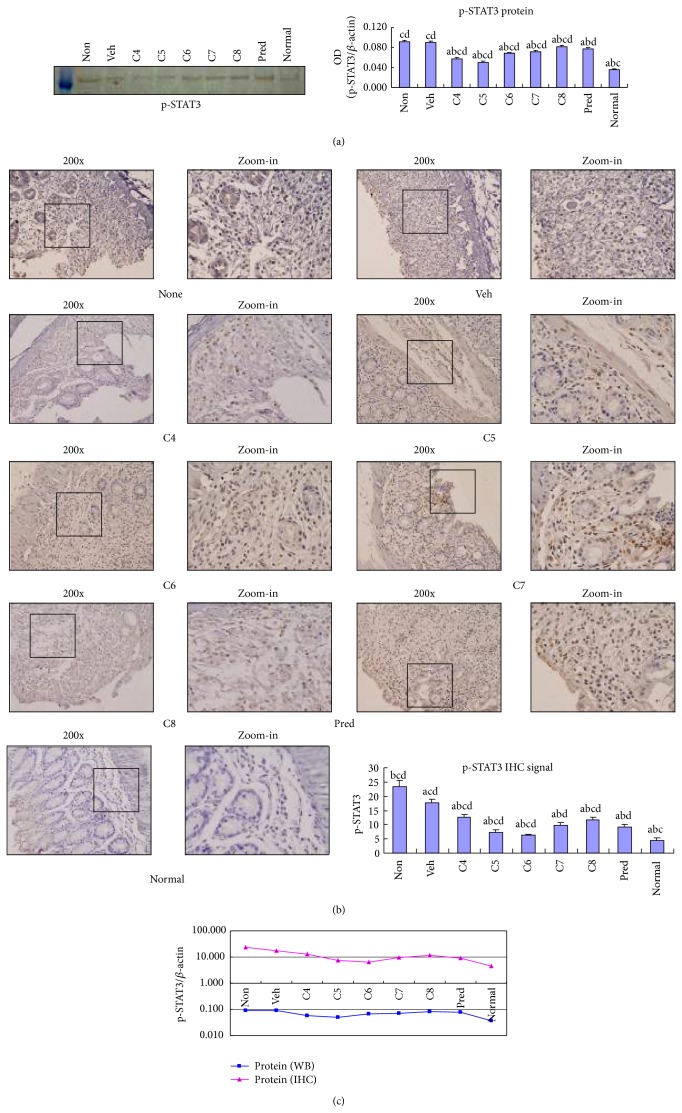
*Lactobacillus acidophilus* treatment attenuated the phosphorylation level of STAT3 (p-STAT3) in colitis. The expression level of p-STAT3 was determined by both Western blotting (WB, (a)) and immunohistochemistry (IHC, (b)) in the colonic tissues of normal control mice group (normal) and DSS-induced model mice groups, including* L. acidophilus*-treated C4–C8 groups, prednisone acetate treated positive control (Pred), and nontreated (Non) and vehicle (Veh) treated control groups. Representative images of Western blotting and immunohistochemical staining showing subcellular localization of p-STAT3 in the colon of nontreated model control mice are shown on the left, and a bar graph representing mean ± SD (*n* = 8) values is shown on the right. A plotted trendline chart shows p-STAT3 expression in each group as determined by WB and IHC (c). ^a^ *P* < 0.05 versus Non, ^b^ *P* < 0.05 versus Veh, ^c^ *P* < 0.05 versus Pred, and ^d^ *P* < 0.05 versus normal.

STAT3 is an important transcription factor that mediates the establishment of Th17 cells in response to IL-23 and TGF*β*1 [15, 16]. We therefore examined whether STAT3 expression is altered with diminished expression of IL-23 and TGF*β*1 upon* L. acidophilus* treatment. We observed that STAT3 expression was upregulated in the colon of DSS-mediated colitis, which is consistent with previous reports [22, 23]. Although the decrease in the STAT3 mRNA level was relatively moderate in the* L. acidophilus-*treated groups (Figure 5(a)), an apparent decrease in STAT3 protein level was observed in contrast to the vehicle treated control group (Figure 5(b)). Among all the* L. acidophilus*-treated groups, inhibition of STAT3 protein expression was most obvious in C5 (Figure 5(c)). Because phospho- (p-) STAT3 is the functional form, it is important to understand whether the phosphorylation level of STAT3 is altered by* L. acidophilus* treatment. We observed that p-STAT3 was significantly (*P* < 0.05) lower in all groups (C4–C8) relative to the untreated or vehicle treated control groups by Western blotting analysis (Figure 6(a)). Moreover, immunohistochemical staining showed that p-STAT3 localized in the nuclei was predominantly expressed in the inflamed area in the DSS-induced untreated control group (Figure 6(b)). Importantly,* L. acidophilus* treatment caused a significant (*P* < 0.05) reduction in p-STAT3 signal, with a stronger effect in the C5 and C6 groups (Figures 6(b) and 6(c)). These findings suggest that administration of* L. acidophilus* improves colitis by restoring the homeostasis of p-STAT3-mediated cellular events, such as Th17 differentiation and IL-17 secretion.

## 4. Discussion

IBDs, including UC and CD, are considered a consequence of aberrant immune response in the gastrointestinal tract. It is traditionally recognized that CD is associated with Th1 cytokines and UC is modulated by Th2 cytokines. This concept has recently been challenged by the discovery of novel subset Th17 cells. Although controversy remains, Th17 cells are generally accepted as the mediators of intestinal inflammation via the secretion of proinflammatory cytokines, such as IL-17 [22, 23]. Therefore, targeting Th17 cells can be a potential approach for treating IBD. With the accumulation of evidence supporting the significant role of microbiota in gut homeostasis [24, 25], probiotics have nowadays been more and more frequently used in treating IBD, especially UC, through induction of remission and prevention of pouchitis. However, whether and how probiotics modulate Th17 cell fate in the gut remains poorly understood.

We found the expression of IL-17, a hallmark cytokine characterizing Th17 cells, was dramatically increased in colitis, which is consistent with previous reports [7, 8, 21]. Moreover, we herein provide the first evidence that oral administration of* L. acidophilus*, one of the most common natural inhabitants of the human gut, suppressed the upregulation of the proinflammatory cytokine IL-17 in colitis. Our finding is supported by a recent observation by Jan et al. that oral administration of* L. gasseri* suppresses IL-17 induction in allergen-induced airway inflammation in mice [26]. More recently, Amdekar et al. showed that administration of* L. acidophilus* downregulates the expression of IL-17 and to a lesser extent that of TNF*α* in rats with collagen-induced arthritis [27]. Similarly, we also observed an inhibitory effect of* L. acidophilus* on colitis-mediated TNF*α* expression in mouse colon. The decrease in IL-17 expression might be an important contributor to the ameliorative effect of* L. acidophilus* on the pathogenesis of colitis as observed in our previous report [20], because inhibition of IL-17 function with a chemical inhibitor or anti-IL-17 antibody has been extensively demonstrated to be effective in IBD treatment [28–30].

The observed downregulation of IL-17 further indicates that administration of* L. acidophilus* may have modulated the fate of Th17 cells. In inflammation, Th17 cell differentiation is induced by paracrine signaling and Th17 cells are further expanded to achieve full involvement [14]. Accumulating evidence demonstrates that TGF*β*1 is critically involved in Th17 cell differentiation, whereas IL-23 plays an essential role in maintaining proliferation and survival of Th17 cells. Moreover, recent studies have shown that the expression levels of TGF*β*1 and IL-23 are both enhanced in IBD [31–33], thereby promoting the progression of inflammation by activating Th17 differentiation and function. In this study, we showed for the first time that* L. acidophilus* inhibits the colitis-mediated increase in TGF*β*1 and IL-23 expression in mouse colon. These results implicate a decrease in TGF*β*1 and IL-23 expression upon* L. acidophilus* treatment might alter Th17 cell population. However, this suspection needs be testified by immunohistochemical staining of Th17 cell-specific marker, such as IL-17, in colitis treated with and without* L. acidophilus*. Although we did not attempt to elucidate the molecular mechanisms that mediate the inhibitory role of* L. acidophilus* in IL-23 regulation in the present study, we suspect that reduced expression of TNF*α* may be responsible. In fact, previous studies have clearly shown that nuclear factor- (NF-) *κ*B, a critical mediator of TNF*α* signaling, regulates the transcription of the IL-23p19 gene [34]. A recent finding by the de Vrese lab showed that probiotics* Bifidobacterium breve* and* Lactobacillus rhamnosus* GG (LGG) inhibit lipopolysaccharide- (LPS-) activated expression of IL-23 in cultured intestinal cells via inhibition of histone acetylation and enhancement of DNA methylation [35], providing another potential mechanism for* L. acidophilus*-mediated downregulation of IL-23.

The results of this study further indicate that the efficacy of different concentrations of commensal probiotics on the expression of proinflammatory cytokines and relevant effectors must be carefully determined. We found that the greatest inhibitory effect was achieved in the C5 or C6 group depending on the molecule examined, which is largely consistent with our earlier observation that the optimal therapeutic effect against disease activity was from the C6 group [20]. Probiotic overdose might break the homeostasis of the gut community and cause aberrant immune response of the gastrointestinal defense system and thus altered expression of cytokine profiles.

In conclusion, we showed that oral administration of* L. acidophilus* suppressed colitis-associated hyper-response of the IL-23/Th17 axis. Specifically,* L. acidophilus* treatment inhibited the secretion of proinflammatory cytokine IL-17 by downregulating IL-23 and TGF*β*1, which are required for Th17 cell differentiation and stabilization. These findings indicate that the therapeutic role of* L. acidophilus* in IBD treatment, at least in part, involves modulating the IL-23/Th17 immune axis.

## References

[1] Chen Q.-Q., Yan L., Wang C.-Z. (2013). Mesenchymal stem cells alleviate TNBS-induced colitis by modulating inflammatory and autoimmune responses. *World Journal of Gastroenterology*.

[2] Geremia A., Biancheri P., Allan P., Corazza G. R., Di Sabatino A. (2014). Innate and adaptive immunity in inflammatory bowel disease. *Autoimmunity Reviews*.

[3] Zenewicz L. A., Antov A., Flavell R. A. (2009). CD4 T-cell differentiation and inflammatory bowel disease. *Trends in Molecular Medicine*.

[4] Mennechet F. J. D., Kasper L. H., Rachinel N., Li W., Vandewalle A., Buzoni-Gatel D. (2002). Lamina propria CD^4+^ T lymphocytes synergize with murine intestinal epithelial cells to enhance proinflammatory response against an intracellular pathogen. *The Journal of Immunology*.

[5] Rossi M., Bot A. (2013). The Th17 cell population and the immune homeostasis of the gastrointestinal tract. *International Reviews of Immunology*.

[6] Troncone E., Marafini I., Pallone F., Monteleone G. (2013). Th17 cytokines in inflammatory bowel diseases: discerning the good from the bad. *International Reviews of Immunology*.

[7] Hölttä V., Klemetti P., Salo H. M. (2013). Interleukin-17 immunity in pediatric crohn disease and ulcerative colitis. *Journal of Pediatric Gastroenterology and Nutrition*.

[8] Ueno A., Jijon H., Chan R. (2013). Increased prevalence of circulating novel IL-17 secreting Foxp3 expressing CD4+ T cells and defective suppressive function of circulating Foxp3+ regulatory cells support plasticity between Th17 and regulatory T cells in inflammatory bowel disease patients. *Inflammatory Bowel Diseases*.

[9] Korn T., Bettelli E., Oukka M., Kuchroo V. K. (2009). IL-17 and Th17 cells. *Annual Review of Immunology*.

[10] Zeng X., Wei Y. L., Huang J. (2012). *γδ* T cells recognize a microbial encoded B cell antigen to initiate a rapid antigen-specific interleukin-17 response. *Immunity*.

[11] Fogli L. K., Sundrud M. S., Goel S. (2013). T cell-derived IL-17 mediates epithelial changes in the airway and drives pulmonary neutrophilia. *Journal of Immunology*.

[12] Jovanovic D. V., di Battista J. A., Martel-Pelletier J. (1998). IL-17 stimulates the production and expression of proinflammatory cytokines, IL-*β* and TNF-*α*, by human macrophages. *Journal of Immunology*.

[13] Liu Z.-J., Yadav P. K., Su J.-L., Wang J.-S., Fei K. (2009). Potential role of Th17 cells in the pathogenesis of in flammatory bowel disease. *World Journal of Gastroenterology*.

[14] Yosef N., Shalek A. K., Gaublomme J. T. (2013). Dynamic regulatory network controlling TH 17 cell differentiation. *Nature*.

[15] Yang X. O., Panopoulos A. D., Nurieva R. (2007). STAT3 regulates cytokine-mediated generation of inflammatory helper T cells. *The Journal of Biological Chemistry*.

[16] Zhou L., Lopes J. E., Chong M. M. W. (2008). TGF-*β*-induced Foxp3 inhibits T_H_17 cell differentiation by antagonizing ROR*γ*t function. *Nature*.

[17] Haines C. J., Chen Y., Blumenschein W. M. (2013). Autoimmune memory T helper 17 cell function and expansion are dependent on interleukin-23. *Cell Reports*.

[18] Stritesky G. L., Yeh N., Kaplan M. H. (2008). IL-23 promotes maintenance but not commitment to the Th17 lineage. *Journal of Immunology*.

[19] Toussirot É. (2012). The IL23/Th17 pathway as a therapeutic target in chronic inflammatory diseases. *Inflammation and Allergy—Drug Targets*.

[20] Chen L.-L., Zou Y.-Y., Lu F.-G., Li F.-J., Lian G.-H. (2013). Efficacy profiles for different concentrations of *Lactobacillus acidophilus* in experimental colitis. *World Journal of Gastroenterology*.

[21] Miyauchi E., Ogita T., Miyamoto J. (2013). Bifidobacterium longum alleviates dextran sulfate sodium-induced colitis by suppressing IL-17A response: involvement of intestinal epithelial costimulatory molecules. *PLoS ONE*.

[22] Siakavellas S. I., Bamias G. (2012). Role of the IL-23/IL-17 axis in Crohn's disease.. *Discovery medicine*.

[23] Sarra M., Pallone F., MacDonald T. T., Monteleone G. (2010). IL-23/IL-17 axis in IBD. *Inflammatory Bowel Diseases*.

[24] Dupaul-Chicoine J., Dagenais M., Saleh M. (2013). Crosstalk between the intestinal microbiota and the innate immune system in intestinal homeostasis and inflammatory bowel disease. *Inflammatory Bowel Diseases*.

[25] Elson C. O., Cong Y. (2012). Host-microbiota interactions in inflammatory bowel disease. *Gut Microbes*.

[26] Jan R. L., Yeh K. C., Hsieh M. H. (2012). Lactobacillus gasseri suppresses Th17 pro-inflammatory response and attenuates allergen-induced airway inflammation in a mouse model of allergic asthma. *British Journal of Nutrition*.

[27] Amdekar S., Singh V., Kumar A., Sharma P., Singh R. (2013). Lactobacillus casei and lactobacillus acidophilus regulate inflammatory pathway and improve antioxidant status in collagen-induced arthritic rats. *Journal of Interferon and Cytokine Research*.

[28] Fitzpatrick L. R., Deml L., Hofmann C. (2010). 4SC-101, a novel immunosuppressive drug, inhibits IL-17 and attenuates colitis in two murine models of inflammatory bowel disease. *Inflammatory Bowel Diseases*.

[29] Leppkes M., Becker C., Ivanov I. I. (2009). ROR*γ*-expressing Th17 cells induce murine chronic intestinal inflammation via redundant effects of IL-17A and IL-17F. *Gastroenterology*.

[30] Fitzpatrick L. R., Small J. S., Doblhofer R., Ammendola A. (2012). Vidofludimus inhibits colonic interleukin-17 and improves hapten-induced colitis in rats by a unique dual mode of action. *Journal of Pharmacology and Experimental Therapeutics*.

[31] del Zotto B., Mumolo G., Pronio A. M., Montesani C., Tersigni R., Boirivant M. (2003). TGF-*β*1 production in inflammatory bowel disease: differing production patterns in Crohn’s disease and ulcerative colitis. *Clinical and Experimental Immunology*.

[32] Babyatsky M. W., Rossiter G., Podolsky D. K. (1996). Expression of transforming growth factors *α* and *β* in colonic mucosa in inflammatory bowel disease. *Gastroenterology*.

[33] Liu Z., Yadav P. K., Xu X. (2011). The increased expression of IL-23 in inflammatory bowel disease promotes intraepithelial and lamina propria lymphocyte inflammatory responses and cytotoxicity. *Journal of Leukocyte Biology*.

[34] Zhang Z., Andoh A., Yasui H. (2005). Interleukin-1beta and tumor necrosis factor-alpha upregulate interleukin-23 subunit p19 gene expression in human colonic subepithelial myofibroblasts. *International Journal of Molecular Medicine*.

[35] Ghadimi D., Helwig U., Schrezenmeir J., Heller K. J., de Vrese M. (2012). Epigenetic imprinting by commensal probiotics inhibits the IL-23/IL-17 axis in an in vitro model of the intestinal mucosal immune system. *Journal of Leukocyte Biology*.

